# A New Serum Biomarker Set to Detect Mild Cognitive Impairment and Alzheimer’s Disease by Peptidome Technology

**DOI:** 10.3233/JAD-191016

**Published:** 2020-01-07

**Authors:** Koji Abe, Jingwei Shang, Xiaowen Shi, Toru Yamashita, Nozomi Hishikawa, Mami Takemoto, Ryuta Morihara, Yumiko Nakano, Yasuyuki Ohta, Kentaro Deguchi, Masaki Ikeda, Yoshio Ikeda, Koichi Okamoto, Mikio Shoji, Masamitsu Takatama, Motohisa Kojo, Takeshi Kuroda, Kenjiro Ono, Noriyuki Kimura, Etsuro Matsubara, Yosuke Osakada, Yosuke Wakutani, Yoshiki Takao, Yasuto Higashi, Kyoichi Asada, Takehito Senga, Lyang-Ja Lee, Kenji Tanaka

**Affiliations:** a Department of Neurology, Okayama University, Okayama, Japan; b Department of Neurology, Okayama City Hospital, Okayama, Japan; c Department of Neurology, Gunma University, Graduate School of Medicine, Maebashi, Japan; d Department of Neurology, Geriatrics Research Institute and Hospital, Maebashi, Japan; e Department of Neurology, Ako Chuo Hospital, Ako, Japan; f Division of Neurology, Department of Medicine, Showa University, School of Medicine, Tokyo, Japan; g Department of Neurology, Faculty of Medicine, Oita University, Oita, Japan; h Department of Neurology, Kurashiki Heisei Hospital, Kurashiki, Japan; i Department of Neurology, Himeji Central Hospital, Himeji, Japan; j Membrane Protein and Ligand Analysis Center, Protosera Inc., Osaka, Japan

**Keywords:** Alzheimer’s disease, biomarker, coagulation, complement, MALDI-TOF, mild cognitive impairment, neuroinflammation, peptidome, plasticity

## Abstract

**Background:**

Because dementia is an emerging problem in the world, biochemical markers of cerebrospinal fluid (CSF) and radio-isotopic analyses are helpful for diagnosing Alzheimer’s disease (AD). Although blood sample is more feasible and plausible than CSF or radiological biomarkers for screening potential AD, measurements of serum amyloid- β (Aβ), plasma tau, and serum antibodies for Aβ_1 - 42_ are not yet well established.

**Objective:**

We aimed to identify a new serum biomarker to detect mild cognitive impairment (MCI) and AD in comparison to cognitively healthy control by a new peptidome technology.

**Methods:**

With only 1.5*μ*l of serum, we examined a new target plate “BLOTCHIP^®^” plus a matrix-assisted laser desorption/ionization time-of-flight mass spectrometry (MALDI-TOF/MS) to discriminate control (*n* = 100), MCI (*n* = 60), and AD (*n* = 99). In some subjects, cognitive Mini-Mental State Examination (MMSE) were compared to positron emission tomography (PET) with Pittsburgh compound B (PiB) and the serum probability of dementia (SPD). The mother proteins of candidate serum peptides were examined in autopsied AD brains.

**Results:**

Apart from Aβ or tau, the present study discovered a new diagnostic 4-peptides-set biomarker for discriminating control, MCI, and AD with 87% of sensitivity and 65% of specificity between control and AD (^***^p < 0.001). MMSE score was well correlated to brain Aβ deposition and to SPD of AD. The mother proteins of the four peptides were upregulated for coagulation, complement, and plasticity (three proteins), and was downregulated for anti-inflammation (one protein) in AD brains.

**Conclusion:**

The present serum biomarker set provides a new, rapid, non-invasive, highly quantitative and low-cost clinical application for dementia screening, and also suggests an alternative pathomechanism of AD for neuroinflammation and neurovascular unit damage.

## INTRODUCTION

Dementia is an emerging problem in the world [1], where Alzheimer’s disease (AD) occupies more than 65% of dementia in the developed countries, followed by mild cognitive impairment (MCI), vascular dementia (VaD), and other types of dementia [2]. Diagnosis of AD is usually based on clinical criteria, but may be supported by biochemical markers such as a decreased amyloid-β (Aβ) 42 [3], and increased Aβ oligomer [4, 5] and tau protein in cerebrospinal fluid (CSF) [3]. Radiological analyses are also sometimes undertaken as supportive biomarkers of AD with single photon emission computed tomography (SPECT) for evaluating cerebral blood flow [6, 7] and positron emission tomography (PET) for Aβ using Pittsburgh compound B (PiB) [8–10] and tau [11–13]. However, blood samples are more feasible and plausible than CSF or radiological biomarkers for screening emerging number of potential AD and other dementias [14–16]. Several approaches have been reported for measuring serum Aβ [17, 18], plasma phosphorylated-tau [19], and serum miRNA-455-3p [20]. Serum levels of specific antibodies for Aβ_1 - 42_ monomer and soluble oligomer were not different among normal control, MCI, and AD [21].

Matrix-assisted laser desorption/ionization time-of-flight mass spectrometry (MALDI-TOF/MS) technology can detect small- to medium-sized peptides (1,000–10,000 Da or 10–100 amino acids) in the serum. However, most previous peptidomic analyses required the removal of large amounts of plasma proteins with variable methods before the step of MS analysis, which overlooked hundreds of potentially important endogenous peptides that bind the plasma proteins [22, 23]. In order to compensate such drawbacks of previous peptidomic methodologies, we successfully developed a one-step direct transfer technology using a new target plate “BLOTCHIP^®^” before MALDI-TOF/MS analysis [24]. Furthermore, this new BLOTCHIP^®^-MS technology enabled the comprehensive investigation of serum peptides without missing protein-binding peptides, and thus provided a high throughput capacity for discovery of new peptide biomarkers [24]. The aim of the present study was, therefore, to newly discover and validate serum biomarker peptides and to demonstrate the potential usefulness of these candidate peptides for dementia diagnosis.

## MATERIALS AND METHODS

### Participants and serum

In the present study, participants were prospectively collected at the multicenter, and divided into three groups of cognitively normal control, MCI subject due to AD, and AD patients depending on cognitive function with respect to gender- and age-matching. Cognitive function was examined with Clinical Dementia Rating (CDR) [25, 26] and Mini-Mental State Examination (MMSE) [27]. The participants were clinically evaluated to be cognitively normal, MCI, or AD based on the NINDS-ADRDA criteria [28] or a diagnostic MCI entity [29]. From these participants, 8 ml of blood were collected into a glass test tube (Venoject II, Termo, Japan), which was placed for 1 h at room temperature (RT), and was centrifuged at 1,000 g for 10 min at RT. Resultant supernatant (serum) was divided into 4 Eppendorf tubes (1 ml each) and temporarily stored at –80°C until examination.

### BLOTCHIP^®^-MS analysis

Serum peptidomic analysis was conducted by newly established one-step direct transfer technology “BLOTCHIP^®^-MS analysis”, a rapid quantitative technology for peptidomic analysis [24]. Serum samples (each 1.5*μ*l) were subjected to 4–12% gradient sodium dodecyl sulfate (SDS)-polyacrylamide gel electrophoresis to separate peptides far from proteins. Next, peptides in the gel were electroblotted onto BLOTCHIP^®^ (Protosera Inc., Osaka, Japan). MALDI matrix, *α*-cyano-4-hydroxycinnamic acid (CHCA) (Sigma-Aldrich Co., MO, USA), was applied directly onto BLOTCHIP^®^, and peptidome profiles were obtained in a linear mode of ultrafleXtreme TOF/TOF (Bruker Daltonics Inc. MA, USA), as previously described in detail [30]. All sample measurements were repeated 4 times.

All statistical analyses of MS spectral data were conducted using ClinProTools version 3.0 (Bruker Daltonics). MS spectra obtained were baseline-subtracted, normalized, recalibrated, and peak-picked within the software. Peak heights, which showed significant statistical differences between 2 groups (control subjects versus AD patients), were analyzed using the Wilcoxon test, a nonparametric test for 2-group comparisons. A probability of *p* < 0.05 was considered statistically significant.

### Identification of candidate peptides

Several sera (each 500*μ*l) containing high amount of each peptide were mixed for candidate peptide identification. Peptides were extracted using a Sep-Pak C18 solid-phase extraction cartridge (Waters Corporation, Milford, MA, USA) with 80% (v/v) acetonitrile (ACN) in water containing 0.1% trifluoroacetic acid (TFA), and the eluent was concentrated up to 100*μ*L using a CC-105 centrifugal concentrator (TOMY SEIKO Co, Ltd., Tokyo, Japan). Next, the solution was diluted with 400*μ*L of 2% (v/v) ACN in water containing 0.065% TFA (eluent A) and applied to an ÄKTA purifier (GE Healthcare UK Ltd, Buckinghamshire, England) equipped with a C18 silica-based column (XBridge Shield RP18 2.5 mL, 4.6 mmI.D.*150 mm, Waters Corporation). The eluate was fractionated into 20 fractions (1 mL each) by a liner gradient of 0–100% of 80% (v/v) ACN in water containing 0.05% TFA against eluent A at a flow rate of 1.0 mL/min. Each fraction was concentrated using a CC-105 centrifugal concentrator up to 10*μ*L.

Then the peptide sequences were analyzed using MALDI-TOF/TOF (ultrafleXtreme TOF/TOF) and LC-MS/MS (Fusion; Thermo Fisher Scientific Inc., Waltham, MA, USA). MALDI-TOF/MS was used for smaller peptide with molecular weight (MW) less than 3,500 Da, and LC-MS/MS for larger peptide with MW of 3,500 Da or more. In search of MALDI-TOF/MS data, MASCOT software was used for a “MS/MS ions search”. Parent peptide and MS/MS ions tolerance parameters were set at±100 ppm and±0.7 Da, respectively. In search of LC-MS/MS data, MASCOT program was used (Matrix Science Inc., Boston, USA). Parent peptide and MS/MS tolerance parameters were set at±0.02–0.1 Da and±0.1–0.6 Da. Since relatively large peptides were analyzed with LC-MS/MS, the value of the parent peptide tolerance (±0.02–0.1 Da) was set to allow unanticipated modifications in the sequence. Swiss-Prot sequence database, of which taxonomy was limited to “human”, was selected for the searches. “oxidation”, “phosphorylation”, “N-acetylation”, and “C-cysteinylation” were selected as variable modifications.

### PET imaging

Separately from the above serum analysis, 11 subjects (1 control, 3 MCI, and 7 AD) participated Aβ imaging with Pittsburgh compound B (PiB) detected by positron emission tomography (PET). ^18^F-labeled PiB (Florbetapir) was synthesized with NEPTIS^®^ plug-01, intravenously injected for 9 participants at an Okayama University-affiliated hospital, and 60–90 min later PET images were detected for 30 min. For two AD patients at Oita University, ^11^C-PiB was injected to take PET images [9, 10]. As the whole cerebellar region of interest (ROI) for reference, an average of standard uptake value (SUV) from 6 cerebral cortical areas were calculated in order to analyze positive or negative for PiB-PET (positive for more than 1, negative for 1 or less). The sera of these 11 subjects were also measured for BLOTCHIP^®^-MS analysis.

### Immunohistochemistry for human brain

After candidate serum peptides were detected, corresponding protein expressions were examined in AD brain sections. Five cases of pathologically-proven AD brains and 6 cases of control brains were fixed with formalin, embedded in paraffin, and cut on microtome in 5*μ*m thickness. For Nissl staining, the sections were incubated in 0.1% cresyl violet for 5 min at room temperature, dehydrated gradually in ethanol, and coverslipped with micro cover glass. For single immunohistochemistry, brain sections were dewaxed in xylene and were hydrated in graded ethyl alcohol (100%, 95%, 80%, 70%, 50%) and then distilled water. For antigen retrieval, sections were microwaved in boiling 10 mM citric acid buffers of pH 6. After boiling again, sections were cooled at room temperature for 20 min prior to processing for immunohistochemistry. After incubation in 0.3% hydrogen peroxide/methanol followed by bovine serum albumin, the sections were stained overnight at 4°C with the following primary antibodies: rabbit anti-fibrinogen β chain (FBC) antibody (1:2500, Sigma, St. Louis, MO); rabbit anti-alpha-2-HS-glycoprotein (AHSG) antibody (1:50, Cloud-Clone Corp, Houston, TX, USA); rabbit anti-fibrinogen *α* chain (FAC) antibody (1:125, Sigma, St. Louis, MO); rabbit anti-plasma protease C1 Inhibitor (PPC1I) antibody (1:50, Proteintech Group, Chicago, IL). Brain sections were then washed with PBS and treated with suitable biotinylated secondary antibodies (1:500; Vector Laboratories, Burlingame, CA) for 2 h at room temperature. The slides were then treated with avidin-biotin-peroxidase complex (Vectastain ABC Kit; Vector) for 30 min and incubated with diaminobenzidine tetrahydrochloride (DAB). As for the negative control, we stained a set of brain sections in the same manner without the primary antibody. A light microscope (Olympus BX-51, Tokyo, Japan) was used to examine the sections.

For each measurement, we analyzed four randomly selected regions in each section. For the semiquantitative analysis, the number of FBC, AHSG, FAC, and PPC1I-positive cells were calculated in the cerebral cortex and hippocampus (HI).

### Statistical analysis

Diagnostic performance of the peptides was evaluated using R statistical computing environment software [31]. Receiver operating characteristic (ROC) analysis was performed with package ‘Epi’ [32] within R software. Areas under the curve (AUC) values were calculated from ROC curve as an indicator of the diagnostic value. The optimal cutoff thresholds for diagnosis were determined according to Youden’s index [33]. Multiple binomial logistic regression analysis of peptides was conducted for detection of the best combination of peptides discriminating the two groups using R package ‘Aod’. Relative PET value was calculated to be positive as above 1, and negative as 1 or less. Correlation coefficient was also calculated by Pearson product-moment correlation coefficient between MMSE and relative PET value or serum probability of dementia (SPD). Immunohistochemical data were analyzed in GraphPad Prism (version 7.0, GraphPad Software Inc., San Diego, CA, SCR_002798) and presented as mean±SD. Two-way analysis of ANOVA was used to examine the differences in the expression of immunohistochemistry analysis between groups and brain areas followed by Sidak’s multiple comparisons test. In all statistical analyses, data with *p* < 0.05 were considered to be significant.

The present study was approved by the Ethical Committee of Graduate School of Medicine, Dentistry and Pharmaceutical Science, Okayama University (#OKU-1603-031 = peptidome, #OKU-1709-004 = PET, #OKU-1904-019 = pathology), Gunma University (CIRU-1665), and Oita University (B12-013).

## RESULTS

### Participants and candidate peptides

Totally 259 participants were collected for the present study, consisted of 100 normal control subjects, 60 MCI due to AD, and 99 AD patients (Table 1). Mean ages of these three groups were 80.0–81.9 years old, that were not significantly different. Mean MMSE were 28.6±1.5, 26.0±2.5 (^**^p < 0.01 versus control), and 18.4±4.9 (^**^p < 0.01 versus control, ^# #^*p* < 0.01 versus MCI) in control, MCI and AD, respectively. Among these three groups, four peptides were identified to show significantly different between control versus MCI, MCI versus AD, and control versus AD (Table 2). The four peptides were 27 amino acid fragment (Peptide #1) of fibrinogen β chain (FBC), 27 amino acid fragment (Peptide #2) of *α*2-HS-glycoprotein (AHSG), 47 amino acid fragment (Peptide #3) of fibrinogen *α* chain (FAC), and 34 amino acid fragment (Peptide #4) of plasma protease C1 inhibitor (PPC1I) (details in Table 2).

**Table 1:** Participants summary of the present study

	Normal control	MCI due to AD	AD patients
No. of subjects	*n* = 100	*n* = 60	*n* = 99
Female gender	60%	43%	56%
Age (y)	80.0±3.9	80.9±3.7	81.9±3.9
MMSE	28.6±1.5	26.0±2.5^**^	18.4±4.9^**,^^# #^

Data are expressed mean±SD. ^**^p < 0.01 versus control, ^##^*p* < 0.01 versus MCI.

**Table 2:** List of 4 identified peptides

Peptide #	Mother protein (abbreviation)	Calculated monoisotopic mass [M+H]^+^	Amino acid number (N-/C- terminus)	Swiss-Prot accession number	Peptide sequence
1	Fibrinogen β chain (FBC)	2882.54	27 (45–71)	P02675	GHRPLDKKREEAPSLRPAPPPISGGGY
2	*α*2-HS-glycoprotein (AHSG)	2858.53	27 (341–367)	P02765	TVVQPSVGAAAGPVVPP**C**PGRIRHFKV (C18 = cysteinylation)
3	Fibrinogen *α* chain (FAC)	5078.35	47 (528–574)	P02671	TF**P**GFFSPMLGEFVSETESRGSESGIFTNTK ESSSHHPGIAEFPSRG (P3 = oxidation)
4	Plasma protease C1 inhibitor (PPC1I)	4151.17	34 (467–500)	P05155	TLLVFEVQQPFLFVLWDQQHKF PVFMGRVYDPRA

### Diagnostic performances of four peptides

Diagnostic performance of single marker peptide is summarized in Table 3, in which AUC was 0.710 for Peptide #1, 0.615 for Peptide #2, 0.616 for Peptide #3, and 0.594 for Peptide #4, respectively, with variable sensitivity (37–83%) and specificity (36–87%). Some of them showed a low sensitivity but high specificity (Peptide #3), and a high sensitivity but low specificity (Peptide #4). Peptide #2 showed a significantly lower fold change (0.84) compared to the three other increases of Peptide #1, #3, and #4 (Table 3). A multiple binomial logistic regression model was constructed by using the four peptides. After the examination of samples of a training data set (100 control subjects and 99 AD patients), an optimized model with the highest diagnostic performance was obtained as follows: Probability = 1/(1+*e*∧(–(–0.5473 + 3.719E-04 [Peptide #1] - 7.584E-05 [Peptide #2] + 1.302E-04 [Peptide #3] + 4.411E-05 [Peptide #4]))) (Equation 1).

**Table 3:** Diagnostic performance of single marker peptide

Peptide #	Mother protein (abbreviation)	AUC	Sensitivity (%)	Specificity (%)	Elecro- signal cut off	Fold change (AD/Control)	*p* (Mann-Whitney’s U test)
1	Fibrinogen β chain (FBC)	0.710	76	58	3,550	1.44	^***^p < 0.001 (3.1×10^–7^)
2	*α*2-HS-glycoprotein (AHSG)	0.615	62	59	28,274	0.84	^**^p < 0.01 (0.005)
3	Fibrinogen *α* chain (FAC)	0.616	37	87	7,995	1.16	^**^p < 0.01 (0.005)
4	Plasma protease C1 inhibitor (PPC1I)	0.594	83	36	1,886	1.60	^*^p < 0.05 (0.021)

With this 4-peptide multi-marker set (Table 4), AUC of control versus MCI was 0.662, of MCI versus AD 0.672, and of control versus AD 0.804 with providing high sensitivity (72–87%) and high specificity (59–65%). To assess validity of the logistic model, k-fold cross validation was conducted. In k-fold cross-validation, the dataset was randomly divided into k subsets with equal size for each group. In this study, k = 5 was chosen. Logistic model using multiple peptides was trained for 5 times with each time leaving out one of the subsets from training. The omitted subset was applied for calculation of the diagnostic values, e.g., AUC. These values were compared with the diagnostic performance of the logistic regression model. By using 5-fold cross validation, AUC of control versus AD was estimated to be 0.796, which was close to the value without cross-validation, 0.804. Fold changes of MCI/control, AD/MCI, and AD/control were 1.39, 1.37, and 1.90, respectively (Table 4).

**Table 4:** Diagnostic performance of the four-peptide multi-marker set

Sample data	AUC	Sensitivity (%)	Specificity (%)	Probability cut off	Fold change	*p* (Mann-Whitney’s U test)
Control versus MCI	0.662	72	59	0.357	MCI/Control = 1.39	^***^p < 0.001 (6.2×10^–4^)
MCI versus AD	0.672	77	62	0.477	AD/MCI = 1.37	^***^p < 0.001 (2.8×10^–4^)
Control versus AD	0.804	87	65	0.385	AD/Control = 1.90	^***^p < 0.001 (1.3×10^–13^)

**Fig.1:**
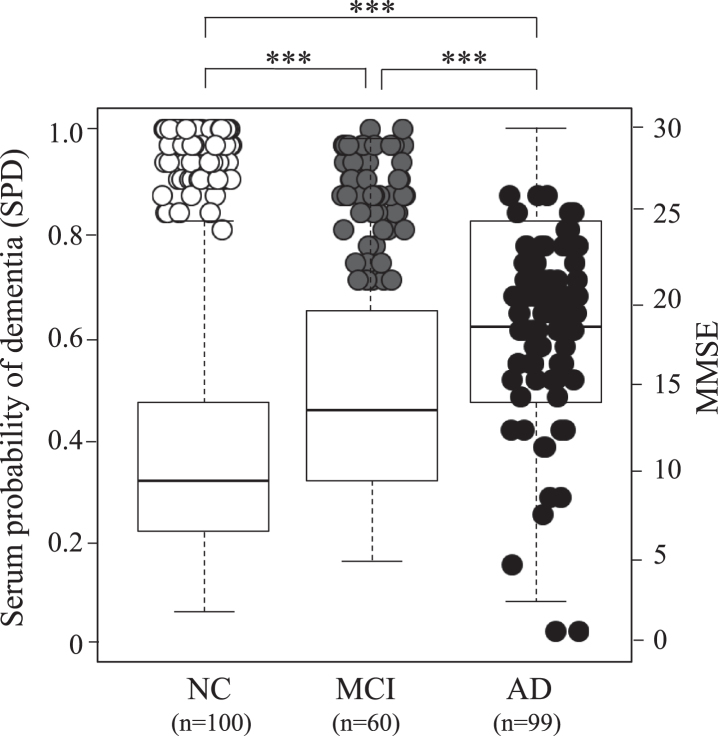
Serum probability of dementia (SPD, boxes) and MMSE (circles) in 100 normal control (NC) subjects, 60 MCI due to AD subjects, and 99 AD patients. Note serial increase of SPD and decrease of MMSE from NC, MCI to AD. Open circle represent NC, grey MCI, and black AD.

Figure 1 shows SPD depending on the peptide data among the three groups, which is overlaid by 259 cases of MMSE. These three groups show significantly different SPD between control versus MCI, MCI versus AD, and control versus AD (^***^p < 0.001). A good correlation was found in the increase of SPD and the decrease of MMSE among clinically diagnosed three groups (Fig. 1).

### Amyloid PET and brain pathology

**Fig.2:**
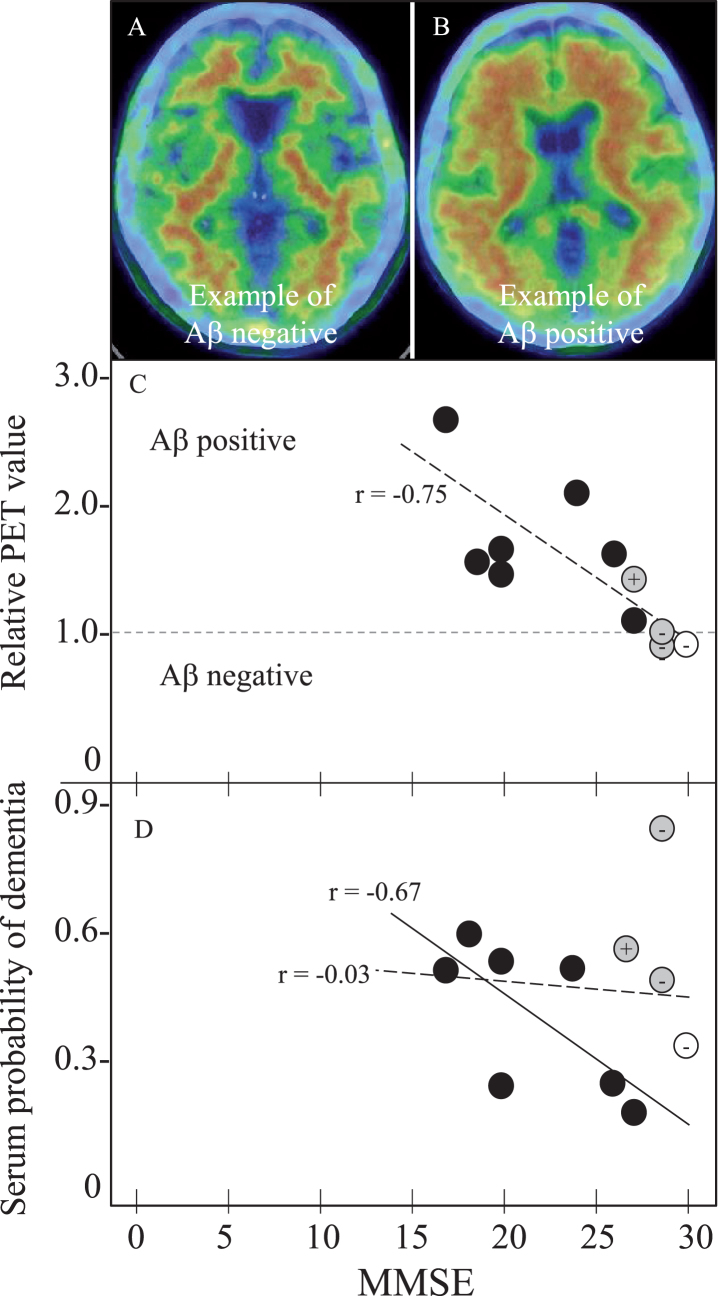
Amyloid PET and SPD with MMSE in 11 PET subjects for 1 NC, 3 MCI, and 7 AD. Panels A and B shows Aβ negative- and positive-example, (C) MMSE versus relative PET value of negative with 1 or less and positive with more than 1, and (D) MMSE versus SPD. Note the cortical Aβ deposit in PET-positive example (B), the strong correlation of relative PET value with MMSE in all 11 subjects (*r* = –0.75, *p* = 0.0070, panel C, oblique dotted line), and a significant correlation of SPD with MMSE in 7 AD patients (*r* = –0.67, *p* = 0.0908, panel D, oblique dotted line). Correlation coefficient of SPD versus MMSE was *r* = –0.03, *p* = 0.9198 for all 11 subjects (D, dotted line). Open circle represents NC, grey MCI, and black AD. + or – of grey MCI and control are Aβ-PET positive or negative, respectively.

Table 5 summarizes SPD data of 11 amyloid PET subjects. One control subject (female) was Aβ-PiB negative, who showed 0.33 SPD. Among three MCI subject (1 male and 2 females), two were Aβ-PiB negative (0.82 and 0.48 SPD) and one was Aβ-PiB positive with 0.55 SPD. Seven AD patients (5 males and 2 females) were all Aβ-PiB positive with SPD of 0.19–0.57. Of note was a case of MCI with high SPD (0.82), who was Aβ-PiB negative MCI with MMSE of 29, but converted into AD with MMSE of 23 in 2 years. Two other MCI cases with moderate SPD of 0.48 (Aβ-PiB negative) and 0.55 (Aβ-PiB positive) kept stable MMSEs for subsequent 3 and 18 months, respectively, until expiring the visit to our hospital. Figure 2 depicts Aβ-PiB PET negative (Fig. 2A) or positive (Fig. 2B) examples, and correlation coefficients of MMSE versus PET data (Fig. 2C) and MMSE versus SPD data (Fig. 2D) of these 11 subjects, showing a strong correlation of MMSE versus PET (*r* = –0.75, *p* = 0.0070) among all 11 subjects (Fig. 2C, oblique dotted line) and a correlation of MMSE versus SPD (*r* = –0.67, *p* = 0.0908) in 7 AD patients (Fig. 2D, oblique solid line). Correlation coefficient of MMSE versus SPD was *r* = –0.03 for all 11 subjects (Fig. 2D, dotted line).

**Table 5:** Relative Aβ-PET value and serum probability of dementia (SPD) in 11 subjects

	Normal control	MCI			AD						
		1	2	3	1	2	3	4	5	6	7
Age (y)	77	78	85	86	68	71	67	78	71	67	56
MMSE (/30)	30	29	29	27	27	20	18	26	20	24	17
PET result	–	–	–	+	+	+	+	+	+	+	+
Relative PET value	0.94	0.94	0.95	1.42	1.06	1.45	1.47	1.53	1.54	1.96	2.53
SPD	0.33	0.82	0.48	0.55	0.19	0.54	0.57	0.24	0.24	0.50	0.49

**Fig.3:**
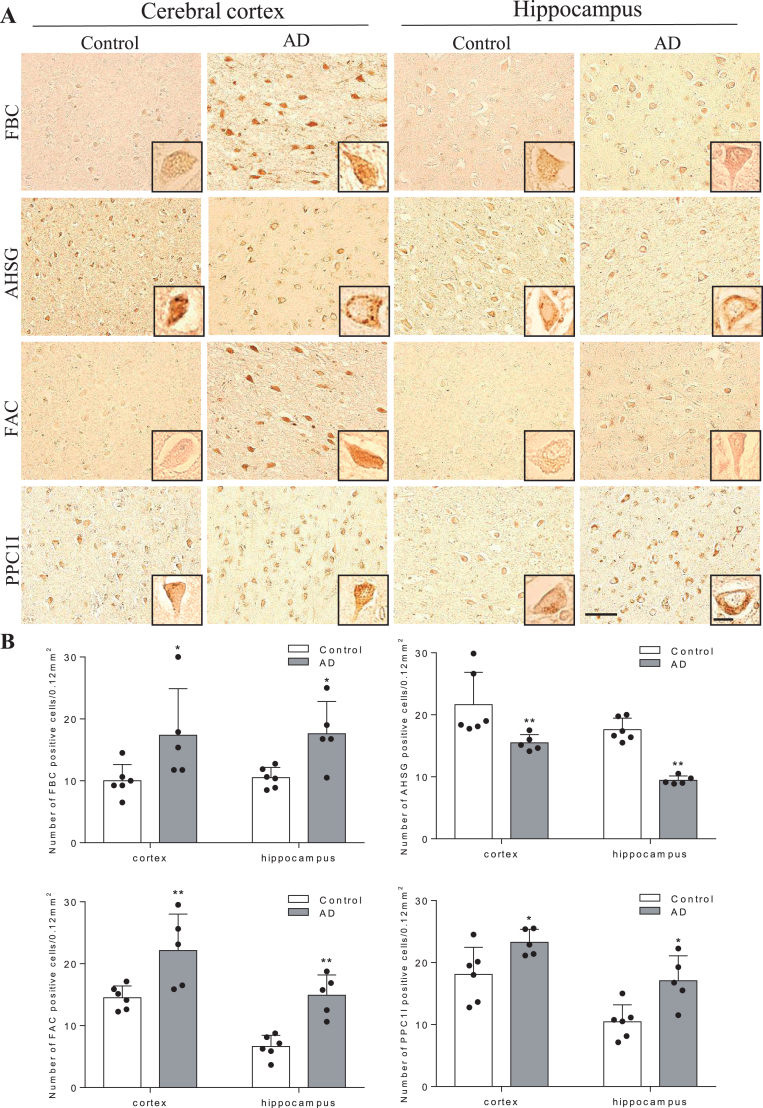
Immunohistochemical pathology of four mother proteins in AD brain. In comparison to control brains, note significant increases of three proteins (FBC, FAC, and PPC1l) and a decrease of AHSG in AD brain. Scale bar = 50*μ*m (^*^p < 0.05 and ^**^p < 0.01 versus Control).

Figure 3 shows histochemical analysis of human brain sections in the cerebral cortex (mainly frontal lobe) and HI. Both FBC and FAC were weakly stained in neurons of cortex and HI of control brains, but were strongly induced in AD brain. AHSG was obviously stained in neurons of cortex and HI of control brains, but was weaker in AD brains. PPC1I was clearly stained in neurons of control brains, which enhanced in AD brains. Quantitative analysis showed significant increases of positive cell numbers in the AD than control brains for FBC, FAC, and PPC1I, but a significant decrease for AHSG (Fig. 3, ^*^p < 0.05 and ^**^p < 0.01 versus Control).

## DISCUSSION

The present study discovered a new serum biomarker with a new peptidome technology. The 4-peptide biomarker set presented a significant difference among age- and gender-matched normal control, MCI, and AD groups with high sensitivity and specificity (Tables 1–4, Fig. 1). Cognitive MMSE score was well correlated to brain Aβ deposition (Fig. 2C) and to SPD of AD (Figs. 1 and 2D), and thus provides a new screening for dementia with a quick, a small amount of serum (1.5*μ*l), a very low invasive, and a low-cost test. The diagnostic performance of each peptide showed a characteristic balance of sensitivity and specificity to discriminate three subject groups (Table 3), each of which mutually compensated to enable a high sensitivity/specificity with high AUC as the set diagnosis (Table 4).

The present new peptidome technology took BLOTCHIP^®^-MS method which omits deproteination step, allowing a quick and whole peptides analysis not only for free peptides but also protein-binding peptides in the serum. This method can pick comprehensive serum peptides without missing protein-binding peptides, that were previously lost after deproteination step. Furthermore, such whole peptides were separated by a simple electrophoresis, and then on-step directly transferred to the high throughput MS analysis. Selection of test tube for blood collection was also very important in the present study. Many hospitals commonly use test tubes which are coated by thrombin to facilitate coagulation and getting serum in a short time. However, our pilot study proved the use of thrombin-coated test tube greatly interfered with the analysis data. Thus, the present study chose a test tube for blood collection that is simply coated by silica (Venoject II), also commonly available in most clinics.

Surprisingly, these peptides were not fragments of Aβ or tau, but were related to coagulation and complement/inflammation systems. The mother protein levels (FBC, FAC, PPC1I) of these three peptides (Peptides #1,3,4) were upregulated and AHSG was downregulated in the human AD patients (Fig. 3). Because frequencies of atrial fibrillation (Af) and the use of anti-coagulative drugs were only between 0 and 4.0% in the control, MCI, and AD groups, the present result is not simply due to the secondary effect of having Af nor the anti-coagulative drug use. FBC and FAC are essential coagulation materials and key contributor of AD pathology [34, 35]. PPC1I is a serpin superfamily, which regulates a pivotal coagulation/neuroinflammation in damaged brain [36, 37], and anti-inflammatory AHSG is regulated under control of pro-inflammatory tumor necrosis factor-*α* [38, 39]. Thus, the present data strongly suggest a new pathomechanism of AD, that is not simple Aβ and tau hypotheses but are in good accordance to our recent reports that suggested a neurovascular unit (NVU) damage and a neuroinflammation/plasticity of AD brain [40–43].

In fact, a recent report suggested an important role of pericyte for maintaining cerebral circulation and pleiotrophin secretion at NVU [44]. Our previous studies also reported that the mother proteins of these peptides (FAC and PPC1I) were upregulated and AHSG was downregulated in AD model mice, which were enhanced by chronic hypoperfusion [38, 45]. These previous mice reports were confirmed in human AD brain samples in the present study (Fig. 3), suggesting the constant activation of coagulation/plasticity and neuroinflammation process both in simple AD and AD plus hypoperfusion brains. In fact, our recent report showed that PiB-PET positive MCI showed elevations of inflammatory cytokines macrophage inflammatory protein-1β and stem cell growth factor-β in CSF [46]. Furthermore, matrix metalloproteinases involve multiple roles as inflammatory components of AD brain [47], which may be detected by a new PET tracer for microglial activation [48]. A recent report showed that Aβ interacted with fibrinogen and induced its oligomerization [49], which may well support the present data.

Increasing numbers of dementia patients demand a simple and quick screening test for early diagnosis. A recent report to detect serum Aβ showed a good correlation to brain Aβ deposition detected by PiB with a combination of immunoprecipitation (IP) plus MALDI-TOF/MS [50]. However, the IP-MS is limited to the measurement of known peptides and is not a popular diagnostic method due to enormous expense and equipment to generate and maintain antibodies. On the other hand, the present BLOTCHIP^®^-MS analysis requires no pretreatment of blood samples, because whole peptides are effectively dissociated from major blood proteins during one-dimensional polyacrylamide gel electrophoresis process. Thus, the present one dimension/MALDI-TOF (1-DE/MS) system eliminates staining, extracting, loading and many other time-consuming steps taken in 2-DE/MS, thereby greatly reducing analysis time while providing high throughput peptidomic analysis.

The present study provides a new diagnostic biomarker set for MCI and AD by a new peptidome technology, but also suggests an important pathomechanism of AD for neuroinflammation and NVU damage relating to coagulation/plasticity, that could develop a new approach for a disease modifying therapy or to prevent a conversion from MCI to AD. A standardized kit for automated quantitative assessment with ProtoKey^®^ assay [51–54] with this 4-peptides set will enable a rapid, non-invasive, highly quantitative and low-cost clinical application in the near future.

The authors thank all the participants of the present study and Ms. Kadota A. for her enormous contribution of serum preparations. The present study was partly supported by a Grant-in-Aid for Scientific Research (B) 17H0419619, (C) 15K0931607, and 17K1082709, and by Grants-in-Aid from the Research Committees (Kaji R, Toda K, and Tsuji S) from the Japan Agency for Medical Research and Development 7211700176, 7211700180, and 7211700095.

Asada K, Senga T, Lee LJ, and Tanaka K provided BLOTCHIP^®^-MALDI-TOF/MS analysis at The Membrane Protein and Ligand Analysis Center, Protosera Inc., Osaka, Japan.

Authors’ disclosures available online (https://www.j-alz.com/manuscript-disclosures/19-1016r1).
